# Mortality due to *Cryptococcus neoformans* and *Cryptococcus gattii* in low-income settings: an autopsy study

**DOI:** 10.1038/s41598-019-43941-w

**Published:** 2019-05-16

**Authors:** Juan Carlos Hurtado, Paola Castillo, Fabiola Fernandes, Mireia Navarro, Lucilia Lovane, Isaac Casas, Llorenç Quintó, Francesc Marco, Dercio Jordao, Mamudo R. Ismail, Cesaltina Lorenzoni, Antonio E. Martinez-Palhares, Luiz Ferreira, Marcus Lacerda, Wuelton Monteiro, Ariadna Sanz, Emilio Letang, Lorena Marimon, Susan Jesri, Anelsio Cossa, Inacio Mandomando, Jordi Vila, Quique Bassat, Jaume Ordi, Clara Menéndez, Carla Carrilho, Miguel J. Martínez

**Affiliations:** 1 ISGlobal, Hospital Clínic - Universitat de Barcelona, Barcelona, Spain; 2 Department of Microbiology, Hospital Clinic of Barcelona, Universitat de Barcelona, Barcelona, Spain; 3 Department of Pathology, Hospital Clinic of Barcelona, Universitat de Barcelona, Barcelona, Spain; 4 Department of Pathology, Maputo Central Hospital, Maputo, Mozambique; 5 Faculty of Medicine, Eduardo Mondlane University, Maputo, Mozambique; 6 Universidade do Estado do Amazonas, Manaus, Amazonas Brazil; 7 Fundação de Medicina Tropical Doutor Heitor Viera Dourado, Manaus, Amazonas Brazil; 8 Instituto de Pesquisas Leônidas & Maria Deane, Fiocruz, Manaus, Brazil; 9 Hospital del Mar. Service of Infectious Diseases, Hospital del Mar, Hospital del Mar Research Institute (IMIM), Barcelona, Spain; 10 Centro de Investigação em Saúde de Manhiça, Maputo, Mozambique; 11 ICREA, Catalan Institution for Research and Advanced Studies, Pg. Lluís Companys 23, 08010 Barcelona, Spain; 12 Pediatric Infectious Diseases Unit, Pediatrics Department, Hospital Sant Joan de Déu (University of Barcelona), Barcelona, Spain; 13 Consorcio de Investigación Biomédica en Red de Epidemiología y Salud Pública (CIBERESP), Madrid, Spain

**Keywords:** Clinical microbiology, Fungal infection

## Abstract

Cryptococcosis is a major opportunistic infection and is one of the leading causes of death in adults living with HIV in sub-Saharan Africa. Recent estimates indicate that more than 130,000 people may die annually of cryptococcal meningitis in this region. Although complete diagnostic autopsy (CDA) is considered the gold standard for determining the cause of death, it is seldom performed in low income settings. In this study, a CDA was performed in 284 deceased patients from Mozambique (n = 223) and Brazil (n = 61). In depth histopathological and microbiological analyses were carried out in all cases dying of cryptococcosis. We determined the cryptococcal species, the molecular and sero-mating types and antifungal susceptibility. We also described the organs affected and reviewed the clinical presentation and patient management. Among the 284 cases included, 17 fatal cryptococcal infections were diagnosed. *Cryptococcus* was responsible for 16 deaths among the 163 HIV-positive patients (10%; 95%CI: 6–15%), including four maternal deaths. One third of the cases corresponded to *C. gattii* (VGI and VGIV molecular types, Bα and Cα strains) and the remaining infections typed were caused by *C. neoformans var. Grubii* (all VNI and Aα strains). The level of pre-mortem clinical suspicion was low (7/17, 41%), and 7/17 patients (41%) died within the first 72 hours of admission. Cryptococcosis was responsible for a significant proportion of AIDS-related mortality. The clinical diagnosis and patient management were inadequate, supporting the need for cryptococcal screening for early detection of the disease. This is the first report of the presence of *C. gattii* infection in Mozambique.

## Introduction

Cryptococcosis is the leading cause of meningitis in adults living with HIV in sub-Saharan Africa^1,2^. In 2008, the number of cryptococcal meningitis (CM) cases in this region was estimated to be 720,000 (range 144,000–1.3 million)^3^. Recent estimates from 2014 indicate that over 160,000 (95%CI 113,600–193,900) cases of CM, including more than 130,000 deaths occurred in sub-Saharan Africa^4^. This significant decrease in the absolute number of cases from 2008 to 2014 seems to be related to the scale-up of effective antiretroviral therapy (ART)^5^. However, the proportion of AIDS-related deaths due to *Cryptococcus* remains similar (around 15%), representing the second most common cause of AIDS-related mortality in adults, after tuberculosis^4^. Cryptococcal infection is believed to be acquired by inhalation of fungal cells from the environment. In immunocompetent hosts, the pathogen can be cleared or establish a latent infection^6^. In immunocompromised patients, *Cryptococcus* may induce pneumonia and its dissemination to the central nervous system (CNS) causes meningitis, the most severe form of the infection, which is fatal without appropriate treatment. In low income countries, the one-year mortality of CM, even in HIV-infected patients in care, has been estimated to be as high as 70%^4^.

Human cryptococcal infections were traditionally attributed to *Cryptococcus neoformans* until *Cryptococcus gattii* was classified as a distinct species by molecular methods in 2002^7^. *Cryptococcus gattii* is further divided into four molecular types (variety gattii; VGI-VGIV) and little is known about *C.gatti* infections in Africa, where this pathogen has been isolated from both clinical and environmental samples^8^. Most VGIV strains have been described in the southern part of Africa, whereas VGI and VGII strains have been reported in central Africa^8^. *C. gattii* infections were thought to occur mainly in the tropics^9^ until 2004, when an outbreak of *C. gattii* in North America was recognized^10^. This outbreak was caused by VGII strains and included the emergence of hypervirulent variants^11^. Current knowledge on the epidemiology and clinical presentation of *C. neoformans* infection is clearly greater than that related to *C. gattii*. *C. neoformans* infections occur predominantly in people infected with HIV or with other immunocompromising conditions, whereas *C. gattii* infections have been mainly described in apparently immunocompetent patients^9^. Autopsy findings reveal that the CNS and the lungs are the organs most frequently affected in disseminated infections^12,13^, which may also affect multiple organs, especially in HIV-infected patients^14^. However, reported autopsy series do not usually include cryptococcal species identification, and therefore, knowledge regarding the histopathology of *C. gattii* remains limited.

In the present study, we determined the *Cryptococcus*-associated mortality of a series of 284 autopsies performed in two hospitals located in high prevalence HIV areas, Mozambique in sub-Saharan Africa and the Brazilian Amazonia. We also analysed the clinical presentation, management, and histopathological and microbiological findings of 17 cases of fatal cryptococcal infection.

## Methods

### Study setting and design

An observational study was performed at the Department of Pathology of the Maputo Central Hospital in Maputo, Mozambique and at the *Fundação de Medicina Tropical Doutor Heitor Vieira Dourado*, in Manaus, Western Brazilian Amazon. The study was approved by the *Comissão Nacional de Ética em Pesquisa* - CONEP (Brazil; Ref. 1.074.304), the Clinical Research Ethics Committee of the Hospital Clinic of Barcelona (Spain; File 2013/8677) and National Bioethics Committee of Mozambique (Mozambique; Ref. 342/CNBS/13), which deemed verbal consent sufficient in this study. All research was performed in accordance with guidelines and regulations of the Montreal Statement on Research Integrity in Cross-Boundary Research Collaborations, and the Singapore Statement on Research Integrity.

From November 2013 to March 2015, complete diagnostic autopsies (CDAs) were performed in 284 deceased patients in the two study sites. Two hundred twenty-three cases were recruited from Mozambique: 169 were adults over 15 years of age (112 women, 57 of whom were maternal deaths, i. e., women dying during pregnancy, partum, post-partum or within 42 days of termination of pregnancy^15^), and 54 were children from 1 month to 15 years of age (37 males and 17 females). Sixty-one cases were recruited from Brazil: 59 adults (38 males and 21 females, including one maternal death) and two children. A description of the study design and inclusion criteria for the cases has been published elsewhere^16–18^. All cases fulfilling the inclusion criteria were included in the study; these were (1) a CDA requested by the clinician as part of the medical evaluation of the patient and; (2) a verbal informed consent to perform the autopsy given by the relatives. Traumatic deaths were excluded. A member of the study staff was tasked with liaising with the families in cases of deaths occurring in the pediatric department, but only after the clinicians had asked for consent for postmortem examination^17^. All the clinical records available regarding admission preceding death of each patient were reviewed and the clinical data were collected in a standardized manner.

### Autopsy procedures and determination of the cause of death

The autopsy procedures and a description of the pathological and microbiological methods used have been reported elsewhere^19,20^ and for Briefly, samples of blood, cerebrospinal fluid (CSF), bone marrow and key organs such as the liver, lungs, CNS, spleen and kidneys, as well as uterus in all women of reproductive age were collected for histopathological and microbiological analysis. The microbiological methods included universal screening for relevant pathogens (e.g. HIV, viral hepatitis, tuberculosis, malaria) and bacterial/fungal culture of autopsy samples, targeted screening depending on the patient’s condition (i.e. screening for *Cryptococcus*, *Toxoplasma gondii* and *Pneumocystis jirovecii* in HIV-infected cases), and additional specific testing according to the histopathological findings. Following the analysis of the CDA samples, a panel composed of a pathologist, a microbiologist, and a clinician with expertise in infectious diseases and epidemiology evaluated all the data (including the clinical information) and assigned the final cause of death.

### Laboratory methods

All cases in which the cause of death was assigned to a cryptococcal infection were further characterized. The histological evaluation included periodic acid–Schiff staining (PAS) of the samples of both lungs, CNS, bone marrow, spleen, liver, kidney and uterus, as well as Grocott-Gomorimethenamine silver staining in all CNS samples. Microbiological evaluation included a specific real time PCR for *Cryptococcus* spp.^21^, which was performed in the samples of both lungs, CNS, bone marrow, spleen, liver and uterus. PCR cycle threshold values >38 were considered negative. Plasma and CSF samples were tested by both real time PCR and the *Cryptococcus* antigen (CrAg) lateral flow assay (LFA) (IMMY Inc., Norman, Oklahoma). Discrimination between *C. neoformans var. grubii*, *C. neoformans var. neoformans*, and *C. gattii* was achieved by amplification of the rRNA intergenic spacer (IGS) region, followed by forward and reverse Sanger sequencing of the amplicons. Identities of the cryptococcal species were assigned based on a >98% match to the IGS sequence of a *Cryptococcus* reference strain (*C. gattii*: CBS 6289, ATCC MYA-4561, CBS 6955, ATCC MYA-4563; *C. neoformans var. grubii*: ATCC MYA-4564, ATCC MYA-4565 and *C. neoformans var. neoformans*: ATCC MYA-4567) as described previously^22^.

Cryptococcal strains isolated from the cultures of the autopsy samples underwent a consensus multi-locus sequence typing scheme for *C. neoformans* and *C. gattii*^23^. In addition, the sero-mating type of these strains was determined by multiplex PCR as described previously^24,25^. An anti-fungogram was performed for each strain using the Sensititre® YeastOne® susceptibility plate (TREK Diagnostic Systems, Thermo Fisher Scientific, Oakwood Village, USA).

### Statistical methods

Descriptive analysis was performed using univariate statistics with means and standard deviations for continuous variables and frequency distributions for categorical variables. All analyses, data manipulation, and implementations were done using Stata MP version 15 (Stata, College Station, TX, USA).

## Results

### Mortality due to cryptococcal infections

Among the 284 deceased patients included in this study, 163 (57%) tested positive for HIV, and a total of 17 (6%) fatal cryptococcal infections were confirmed in the CDA. All the deaths except one occurred in HIV-infected cases. Among the HIV-infected cases, cryptococcosis was responsible for 16 deaths (10%; 95% confidence interval [CI]: 6–15%). Among the Mozambican adults, 109 out of 169 (64%) were HIV-infected, and 11 died of fatal cryptococcal infection (10%). Four of these cases were maternal deaths (three pregnant women and one in the puerperal period), accounting for 11% of the 36 maternal deaths occurring in HIV-infected women. None of the 17 HIV-positive children over one month of age had cryptococcal infection. Thirty-seven out of the 61 (61%) patients from Brazil were HIV-infected, and *Cryptococcus* was responsible for five of these deaths (13%). The only death caused by *Cryptococcus* in an HIV-negative patient occurred in a six-year-old child (infected with *C. gattii)*.

### Clinical presentation and management of fatal cryptococcal infections

The demographic features, HIV status, clinical presentation and management of the 17 patients with fatal cryptococcal infection are summarized in Table 1. The median age was 34 years (range 6–44 years); 11 cases (65%) were men. In 13 out of the 16 (81%) HIV-infected patients, a positive HIV test result was reported in the clinical records and was apparently unknown by the clinician in the other 3 cases. Four out of the 16 (25%) HIV-infected patients were on ART, but the duration of ART was only reported in one case.

**Table 1:** Clinical characteristics and management of fatal cryptococcal infections.

Case	Age (in years), sex and origin	Maternal death	HIV Serology	HIV Viral load (copies/mL)	Main symptoms	Pre-mortem Clinical Diagnoses*	Antimicrobial treatment received during illness	Antifungal treatment	Time from admission to death (days)	ART
1	6; M; MOZ	NA	Negative	NA	Fever, seizure, headache, bilateral exophthalmos	**Cerebral cryptococcosis**, pulmonary tuberculosis not confirmed bacteriologically or histologically, essential (primary) hypertension, hypoglycemia unspecified, sepsis due to *Staphylococcus aureus*	Penicillin, chloramphenicol, cephalosporin, quinolones, acyclovir	No	52.3	No
2	30; F; MOZ	Yes	Positive	28,600	Fever, vomits, headache, behavioral changes	HIV disease resulting in encephalopathy, unspecified HIV disease, pneumonia, organism unspecified	Co-trimoxazole, penicillin, cephalosporin	Fluconazole	24.4	Yes 72 months
3	29; F; MOZ	No	Positive	>10,000**	Fever, cough, vomiting, seizure, headache, night sweats, hematemesis	Encephalitis, myelitis and encephalomyelitis, unspecified HIV disease	Cephalosporin, acyclovir	Fluconazole	2.9	NA
4	34; M; MOZ	NA	Positive	7,720	Fever, vomiting, headache, behavioral changes	Encephalitis, myelitis and encephalomyelitis	Cephalosporin	No	1.1	NA
5	36; M; MOZ	NA	Positive	1,180	Headache, exophthalmos, loss of visual acuity	**Cerebral cryptococcosis**, cytomegaloviral disease, unspecified HIV disease, chronic kidney disease, liver failure unspecified	Co-trimoxazole, Cephalosporin	Fluconazole	21.9	NA
6	44; M; MOZ	NA	Positive	8,290	Fever, headache, behavioral changes	Hypertensive encephalopathy, acute renal failure	None	No	4.4	No
7	43; M; MOZ	NA	Positive	21,100	Seizure, headache	Encephalitis, myelitis and encephalomyelitis, unspecified HIV disease	Cephalosporin	No	0.4	Yes
8	25; M; MOZ	NA	Positive	>1,000**	Fever, dyspnea, diarrhea, headache, abdominal pain, melena	Severe and complicated *Plasmodium falciparum* malaria, gastroenteritis and colitis of infectious and unspecified origin	Cephalosporin, quinolone, metronidazole, albendazole	No	0.3	NA
9	35; F; MOZ	No	Positive	18,500	Fever, headache, visual hallucinations, incoherent speech	**Cerebral cryptococcosis**, unspecified HIV disease	Co-trimoxazole, cephalosporin	Amphotericin B	3.2	No
10	21; F; MOZ	Yes	Positive	>100**	Dyspnea	Pneumocystosis, fetal death of unspecified cause, anemia unspecified, unspecified HIV disease, pre-eclampsia	Co-trimoxazole, cephalosporin, metronidazole	No	0.9	NA
11	34; F; MOZ	Yes	Positive	>10,000**	Fever, dyspnea, vomiting, headache	**Cryptococcal meningitis**, unspecified HIV disease, severe acute respiratory syndrome, unspecified hypoglycaemia	Co-trimoxazole, cephalosporin	Amphotericin B	2.8	NA
12	31; F; MOZ	Yes	Positive	>10,000**	Dyspnea, uterine bleeding	Pulmonary edema, unspecified HIV disease; other complications of labor and delivery	Data not available	No	0.0	Yes
13	28; M; BRA	NA	Positive	263	Dyspnea, diarrhea, vomiting, abdominal pain	Unspecified HIV disease, pulmonary tuberculosis confirmed by sputum microscopy with or without culture, other gastroenteritis and colitis of infectious and unspecified origin, sepsis, unspecified	Quinolones, metronidazole, meropenem	No	5.9	Yes
14	32; M; BRA	NA	Positive	NT	Vomiting, headache	Unspecified HIV disease, **cerebral cryptococcosis**	Macrolides, co-trimoxazole	No	12.7	No
15	43; M; BRA	NA	Positive	1,020	Diarrhea, vomiting, headache, asthenia	Unspecified HIV disease, pulmonary tuberculosis, confirmed by unspecified means, **cerebral cryptococcosis**, gastrointestinal hemorrhage, unspecified	None	Fluconazole	4.7	No
16	38; M; BRA	NA	Positive	574,000	Headache	Unspecified HIV disease, **cerebral cryptococcosis**, pneumonia unspecified, respiratory failure unspecified	Macrolides, quinolones, co-trimoxazole	No	15.6	NA
17	34; M; BRA	NA	Positive	17,800	Diarrhea, vomiting, abdominal pain	Unspecified HIV disease, pneumonia unspecified, **cerebral cryptococcosis**, acidosis	Macrolides	Amphotericin B	4.1	No

Clinical diagnoses are listed in the order of differential diagnoses included in the clinical charts. Cryptococcosis is identified in bold, when considered in the list of diagnoses. *As written in the clinical charts by clinicians in charge of patients; M: male; F: female; MOZ: Maputo-Mozambique; BRA: Manaus-Brazil; ART: antiretroviral therapy; NA: not applicable. **The precise quantification was not possible due to the presence of PCR inhibitors in the plasma, which inhibited the amplification of the internal control of the assay. A minimal viral load was inferred from samples with quantifiable viral loads showing a similar cycle threshold for HIV-1 in the PCR assay; NT: not tested due to insufficient amount of sample.

Headache was the most common symptom (13 patients, 76%), followed by fever and vomiting (eight cases each, 47%). Upon admission to hospital, eight patients (47%) were confused and/or agitated, two patients (12%) were lethargic, and another two (12%) were fully comatose. Meningeal signs were detected in seven patients (41%). The mean time from admission to death was 9.3 days (95%CI: 2.4–16.2). Cryptococcal infection was considered the first clinical diagnostic option in only 4/17 (23%) of the confirmed cases, whereas it was included in the differential diagnosis in eight patients (47%). Antifungal treatment (fluconazole or Amphotericin B) had been prescribed to seven patients but to only five of the clinically suspected cases. Seven of the patients (41%) died within 72 hours of admission, and 12 out of the 16 HIV-positive patients (75%) died within one week of admission.

The clinical records of the remaining 267 cases included in this study were reviewed for clinical diagnosis of cryptococcal infection. Among these, three HIV-infected patients were clinically diagnosed with cryptococcal meningitis, but no evidence of cryptococcal infection was found in the autopsy (the cause of death was identified as toxoplasmosis in two cases and tuberculosis in one case).

### Histopathological and microbiological analysis of the fatal cryptococcal infections

Table 2 shows the final cause of death, the cryptococcal species, other associated diagnoses identified in the CDA, as well as the results of the PAS staining, *Cryptococcus* real time PCR and CrAg test in the different samples analysed. Identification of the cryptococcal species was successful in 15 out of the 17 cases. In two cases, the tissue samples provided insufficient DNA to perform IGS amplification and sequencing. Interestingly, among the 15 cases identified, five (33%) were *C. gattii* whereas the remaining cases (66.6%) were *C. neoformans var. grubii*.

**Table 2:** Causes of death, histopathological and microbiological findings.

Case	Cause of death (cryptococcal species)	Other diagnosis/findings	Plasma (PCR Ct value/ CrAg titer)	CSF (PCR Ct value/ CrAg titer)	CNS (PAS/PCR Ct value)	Lung Right (PAS/PCR Ct value)	Lung Left (PAS/PCR Ct value)	Bone marrow (PAS/PCR Ct value)	Liver (PAS/PCR Ct value)	Spleen (PAS/PCR Ct value)	Uterus (PAS/PCR Ct value)	Kidney ** (PAS)
1	Cryptococcal meningoencephalitis (*C. gattii*)	Not relevant	TND /≥1:2560	34.6 /≥1:2560	Positive/31.6	Negative/TND	Negative/TND	Negative/TND	Negative/TND	Negative/TND	NA	Negative
2	Cryptococcal meningoencephalitis (*C. neoformans var. grubii*)	Splenic tuberculosis, esophageal candidiasis	TND /≥1:2560	26.2/≥1:2560	Positive/35.7	Negative/TND	Negative/32.9	Negative/TND	Negative/32.2	Negative/32.9	Negative/36.9	Negative
3	Cryptococcal disseminated disease (*C. neoformans var. grubii*)	Focal cerebral hemorrhages	34.5 /≥1:2560	27.0 /≥1:2560	Positive/31.0	Positive/27.4	Positive/26.7	Positive/27.7	Positive/30.0	Positive/29.8	Positive/NT	Positive
4	Cryptococcal disseminated disease (*C. gattii*)	Past malaria	TND /≥1:2560	26.3 /≥1:2560	Positive/31.3	Positive/30.6	Positive/TND	Negative/TND	Negative/TND	Negative/TND	NA	Negative
5	Cryptococcal disseminated disease (*C. neoformans var. grubii*)	Not relevant	TND /≥1:2560	32.9 /≥1:2560	Positive/36.2	Positive/30.0	Positive/31.6	Positive/36.6	Positive/29.4	Positive/34.3	NT/TND	Positive
6	Cryptococcal disseminated disease (*C. gattii*)	Ischemic cerebrovascular disease, hypertrophic heart, hepatitis	TND /≥1:2560	TND/NT	Positive/33.1	Positive/28.7	Positive/30.8	Negative/TND	Negative/31.5	Negative/32.6	NA	Negative
7	Cryptococcal disseminated disease (*C. gattii)*	Not relevant	NT	29.0/1:160	Positive/34.4	Positive/33.9	Positive/31.6	Negative/37.5	Negative/31.4	Positive/30.5	NA	Negative
8	Cryptococcal disseminated disease (*C. neoformans var. grubii*)	Not relevant	30.8 /≥1:2560	30.0 /≥1:2560	Positive/29.1	Positive/23.4	Positive/24.3	Positive/25.7	Positive/31.8	Positive/22.4	NA	Positive
9	Cryptococcal meningoencephalitis (*C. neoformans var. grubii*)	Not relevant	TND /≥1:2560	23.3 /≥1:2560	Positive/33.5	Positive/29.9	Positive/31.0	Positive/36.1	Negative/29.7	Positive/29.3	NA	Negative
10	Cryptococcal disseminated disease (*C. neoformans var. grubii*)	Not relevant	30.5 /≥1:2560	25.9 /≥1:2560	Positive/24.1	Positive/33.2	Positive/31.0	Positive/29.9	Positive/27.6	Positive/27.4	Positive/28.2	Positive
11	Cryptococcal disseminated disease (*C. neoformans var. grubii*)	Not relevant	30.3 /≥1:2560	31.1 /≥1:2560	Positive/30.8	Positive/27.1	Positive/27.6	Positive/34.8	Positive/30.0	Positive/29.2	NT*/28.5	Positive
12	Cryptococcal disseminated disease (*C. neoformans var. grubii*)	Esophageal candidiasis	35.1 /≥1:2560	TND/1:40	Positive/31.8	Positive/29.7	Positive/29.0	Positive/29.9	Positive/31.0	Positive/28.0	Negative/29.5	Positive
13	Cryptococcal disseminated disease (*C. gattii*)	Tuberculosis	29.9/1:320	NT	Positive/34.6	Positive/28.6	Positive/24.7	Negative/TND	Negative/TND	Negative/31.1	NA	Negative
14	Cryptococcal disseminated disease (*C. neoformans var. grubii*)	Not relevant	NT /≥1:2560	NT /≥1:2560	Positive/22.1	Positive/33.7	Positive/26.6	Negative/33.2	Negative/32.2	Positive/28.9	NA	Positive
15	Cryptococcal disseminated disease (*C. neoformans var. grubii*)	Sepsis due to *Streptococcus pneumoniae*	32.0 /≥1:2560	29.2 /≥1:2560	Positive/31.6	Positive/28.8	Positive/27.6	Positive/28.9	Positive/TND	Positive/26.4	NA	Positive
16	Cryptococcal meningitis (*Cryptococcus spp*.)	Pneumonia (CMV)	TND/1:1280	TND/NT	Positive/31.5	Negative/TND	Negative/30.9	Negative/TND	Negative/TND	Negative/TND	NA	Negative
17	Cryptococcal meningitis (*Cryptococcus spp*.)	Pulmonary histoplasmosis. Toxoplasmosis	TND≥1:2560	31.5/1:1280	Positive/29.7	Negative/TND	Negative/TND	Negative/TND	Negative/TND	Negative/TND	NA	Negative

CNS: Central Nervous system; CSF: cerebrospinal fluid; CoD: cause of death; TND: Target Not Detected; NT: not tested due to insufficient amount of sample; NA: Not applicable; PAS: Periodic acid–Schiff staining; *PAS positive in placental tissue; PCR: Cycle threshold of a specific real time PCR for *Cryptococcus* is indicated; **no kidney samples for PCR testing were collected.

**Figure 1:**
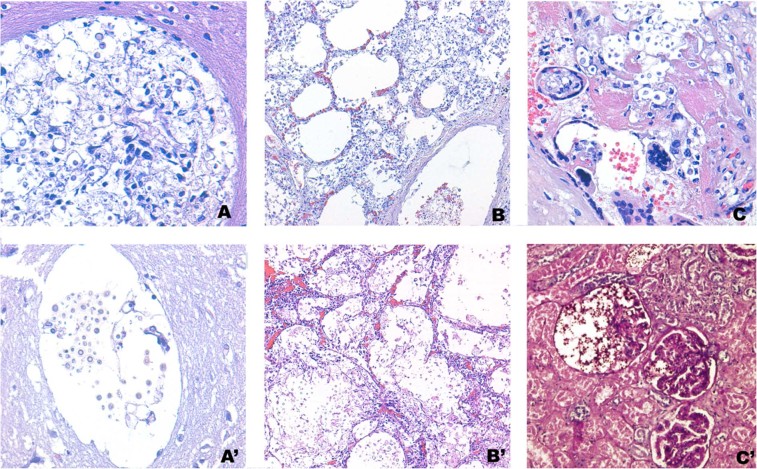
Cryptococcal disseminated infections: (**A**) *Cryptococcus neoformans* infecting the central nervous system (hematoxylin and eosin, 400×). (A’) *Cryptococcus gattii* infecting the central nervous system (hematoxylin and eosin, 400×); abundant capsulated yeasts growing extensively in the perivascular spaces, which become cystically dilated; virtually no inflammatory reaction is seen. (**B**) *Cryptococcus neoformans* infecting the lung (hematoxylin and eosin, 100×); (B’) *Cryptococcus gattii* infecting the lung (hematoxylin and eosin, 100×); abundant capsulated yeasts growing extensively in the alveolar spaces; scant inflammatory reaction is seen. (**C**) *Cryptococcus neoformans* infecting the placenta (hematoxylin and eosin, 400×); (C’) *Cryptococcus neoformans* infecting the kidney (Periodic Acid Schiff (PAS) Stain, 100×).

Twelve of the 17 cases (70%) were diagnosed as disseminated infections and the remaining 5(30%) as meningoencephalitis.The CNS was affected in all 17 cases. The following were the most affected organs (PAS and/or PCR positive): the lungs (88%), spleen (76%), liver (71%), bone marrow (59%) and kidney (47%). In addition, in five out of six (83%) deceased women, the pathogen was detected in uterus samples, and in one case it was detected in the placenta. *Cryptococcus* was found in more than 5 different samples in 11 patients (65% of the cases). More organs were affected in *C. neoformans var. grubii* than in *C. gattii* infections (mean of 6.2 vs. 2.8 organs positive by PAS staining), but no specific histopathological differences were observed between the two *Cryptococcus* species. Molecular testing expanded the detection of *Cryptococcus* in 14 additional tissues that were negative for PAS staining. In contrast, two tissue samples were positive for PAS staining but negative by real time PCR. Periodic acid–Schiff staining and Grocott-Gomorimethenamine silver staining showed identical results. Representative histopathological images of different affected tissues are shown in Fig. 1. *Cryptococcus* was detected by PCR in CSF and plasma in 80% and 47% of the cases tested, respectively. All plasma and CSF samples tested for CrAg LFA were positive. Co-infections with other AIDS-related pathogens or AIDS-defining illnesses (*Mycobacterium tuberculosis*, cytomegalovirus, esophageal candidiasis, *Toxoplasma gondii*, and *Histoplasma capsulatum*) were detected in five cases.

### *Cryptococcus* strain characterization

Eight strains were isolated by culture, seven from the CSF and one from the blood. The molecular characterization and antifungal susceptibility of the cryptococcal strains isolated are shown in Table 3. All *C. neoformans var. grubii* corresponded to the VNI molecular type, whereas two different genotypes, VGI and VGIV, were identified in the two *C. gattii* isolates. Sero-mating type Aα was found in all the *C. neoformans* isolates. Bα and Cα strains were found among the *C. gattii* strains.

**Table 3:** Molecular characterization and antifungal susceptibility of isolated cryptococcal strains.

Study number	Sample	Species	Molecular type	Sero-Mating type	Minimum inhibitory concentration (µg/mL)					
					Amphotericin B	Fluconazole	Voriconazole	Itraconazole	Posaconazole	Flucytosine
2	CSF	*Cryptococcus neoformans var. grubii*	VNI	A α	1	8	0.12	0.12	0.25	8
3	CSF	*Cryptococcus neoformans var. grubii*	VNI	A α	1	4	0.06	0.03	0.06	4
4	CSF	*Cryptococcus gattii*	VGI	B α	1	4	0.12	0.03	0.06	1
7	Blood	*Cryptococcus gattii*	VGIV	C α	1	16	0.25	0.06	0.06	4
8	CSF	*Cryptococcus neoformans var. grubii*	VNI	A α	1	4	0.06	0.06	0.06	4
9	CSF	*Cryptococcus neoformans var. grubii*	VNI	A α	0.5	8	0.06	0.03	0.06	16
10	CSF	*Cryptococcus neoformans var. Grubii*	VNI	A α	1	8	0.06	0.03	0.12	4
11	CSF	*Cryptococcus neoformans var. Grubii*	VNI	A α	0.5	8	0.06	0.06	0.12	4

CSF: cerebrospinal fluid.

Minimum inhibitory concentrations (MICs) of a variety of antifungal agents were determined (Table 3). All triazole antifungal agents (fluconazole, voriconazole, itraconazole and posaconazole) had MICs less than or equal to the defined epidemiological cut-off values (ECVs)^26^. One isolate showed a MIC to flucytosine (16 µg/mL), one dilution higher than the ECV (8 µg/mL). Although all the isolates presented a MIC ≤ 1 µg/mL to amphotericin, the MICs of three *C. neoformans* molecular type VNI (1 µg/mL) and two *C. gattii* (1 µg/mL) were one dilution higher than the defined ECVs (0.5 µg/mL)^27^.

## Discussion

In a large series of nearly 300 autopsies, we performed a thorough histopathological and microbiological analysis of 17 fatal cryptococcal infections, 12 from Mozambique and five from Brazil. Several studies on cryptococcal infection have been carried out in Brazil^14,28^, but there are no data from Mozambique. Indeed, despite being a highly endemic area for HIV, a literature search was unable to find reports describing the burden of this pathogen in this country. Thus, to our knowledge, this is the largest autopsy-based description of fatal cryptococcal infections in Mozambique. Interestingly, albeit being Maputo and Manaus two sites from different countries in terms of climate and income, some of the results obtained were very similar, such as the HIV prevalence in deceased patients (over 60%) and the *Cryptococcus* associated mortality in HIV positive patients. In this study *Cryptococcus* was responsible for 10% of the deaths among HIV-infected patients. This figure is in agreement with current estimates of 15% of AIDS-related deaths due to CM^4^. Although treatment with ART has led to an important reduction in the mortality of HIV-infected patients in sub-Saharan Africa, at present, cryptococcal-related death seems to remain similar to 2008 estimates. In agreement with other autopsy series, HIV-associated cryptococcosis is frequently presented as a disseminated infection^14,28^. Other studies have reported that up to one half of cryptococcal infections are detected in HIV-infected patients receiving ART^29^. In our series, only a quarter of the HIV-positive cases (4/16) were under ART. Unfortunately, the duration of ART was only available in one case. This case had a 6-year history of ART and a high viral load, and therefore, likely represents a treatment failure or defaulted. Other explanations for cryptococcal infection in patients receiving adequate ART include recent treatment initiation in patients with very low CD4 counts (late HIV diagnosis) and cases of immune reconstitution inflammatory syndrome^30^, which might also have been present in our series. Despite progress in ART deployment in sub-Saharan Africa, close to 50% of HIV-infected patients continue presenting to health facilities with advanced HIV disease^31^, thereby being at high risk of cryptococcal infection and death. In this regard, the CrAg can be detected up to three weeks before the onset of CM symptoms^32^ and therefore, screening of asymptomatic HIV patients followed by preemptive antifungal treatment might identify patients at risk of developing the disease and contribute to reducing CM-related mortality^33,34^. In the present study, most cases died within the first week of hospital admission, which is in contrast with other series reporting a mean of two weeks between admission and death^35^. Several factors might have contributed to the rapid fatal outcome in our series. Firstly, the low frequency of clinical suspicion of cryptococcosis may explain the absence of prescribed antifungal treatment in more than half of the patients. On the other hand, some patients may have presented to the hospital with advanced severe stages of cryptococcal infection, considering that seven died within three days of admission (four within 24 hours). The longest hospital admission involved a child who died of *C. gattii* meningitis about two months after hospital admission. *C. gattii* infections are rare in children, and as in the child in this series, the infection often occurs in previously healthy children and presents with CNS involvement^9^. The little knowledge available about *C. gattii* in sub-Saharan Africa has been obtained from studies performed in South Africa. Interestingly, in our study four out of the 12 cryptococcal infections (25%) from Mozambique were due to *C. gattii* infections. Although the numbers are small and should be interpreted with caution, these data contrast with previous findings from South Africa, where *C. gattii* represented only 2.4% of the cryptococcal strains isolated over a two-year period^36^. *C. gattii* infections have mainly been reported in immunocompetent hosts, until studies from South Africa revealed that it affects immunocompromised patients as well^37,38^. It has been reported that the VGI *C. gattii* molecular type is much more likely to affect immunocompetent patients than VGIV^9^. However, both genotypes were detected in HIV-infected patients in our study. All strains of *C. neoformans var. grubii* were molecular type VNI and possessed the sero-mating type Aα, similar to most of the strains described in South Africa. To our knowledge, this is the first report *of C. gattii* in Mozambique, and the Bα and Cα types detected have been previously reported in South Africa^39,40^. Although no clinical breakpoints are available for *Cryptococcus*, antifungal susceptibility testing did not suggest clear resistance patterns, with only MICs one dilution above the ECV being found.

A few isolated autopsy reports of fatal *C. gattii* infections in immunocompetent patients have been reported^41^, which may be explained by the fact that the distinction between *C. gattii* and *C. neoformans* was not performed in many studies. Although the numbers are small, dissemination of the infection seems to be less intense in HIV-infected patients with *C. gattii* compared to *C. neoformans*. Larger studies are needed to better assess the histopathological features of *C. gattii* in HIV-infected patients. Moreover, studies based on post-mortem examinations should be performed to better assess the mortality attributable to specific pathogens in low- and middle-income countries. Accurate mortality data can then impact public health policies, for example, guiding prophylactic and treatment schemes for infectious diseases.

In conclusion, our study highlights the substantial mortality associated with cryptococcal infections among HIV-infected patients, supporting current recommendations^42^ of CrAg screening and preemptive therapy, which is even more relevant in settings in which insufficient pre-mortem clinical suspicion is given to this highly prevalent and life-threatening opportunistic infection.

### Ethics approval and consent to participate

The study protocol received approval of the National Mozambican Ethics Committee (ref.342/CNBS/13) and the Ethics Committee of the Hospital Clinic of Barcelona (Spain; approved, File 2013/8677). MIA and CDA procedures were only conducted after verbal informed consent was provided by the relatives.

## Data Availability

All relevant data are within the paper. Any additional data use and transfer is monitored by ISGlobal’s Data Management and Biostatistics Unit (contact e-mail: ubioesdm@isglobal.org).
